# East and west separation of *Rhipicephalus sanguineus* mitochondrial lineages in the Mediterranean Basin

**DOI:** 10.1186/s13071-017-1985-z

**Published:** 2017-01-23

**Authors:** Sándor Hornok, Attila D. Sándor, Snežana Tomanović, Relja Beck, Gianluca D’Amico, Jenő Kontschán, Nóra Takács, Tamás Görföl, Mohammed Lamine Bendjeddou, Gábor Földvári, Róbert Farkas

**Affiliations:** 1 Department of Parasitology and Zoology, University of Veterinary Medicine, Budapest, Hungary; 2 Department of Parasitology and Parasitic Diseases, University of Agricultural Sciences and Veterinary Medicine, Cluj-Napoca, Romania; 3 Laboratory for Medical Entomology, Centre of Excellence for Food and Vector-Borne Zoonoses, Institute for Medical Research, University of Belgrade, Belgrade, Serbia; 4 Laboratory for Parasitology, Croatian Veterinary Institute, Zagreb, Croatia; 5 Plant Protection Institute, Centre for Agricultural Research, Hungarian Academy of Sciences, Budapest, Hungary; 6 Department of Zoology, Hungarian Natural History Museum, Budapest, Hungary; 7 Ecology of Terrestrial and Aquatic Systems (EcoSTAq), University of Badji Mokhtar, Annaba, Algeria

**Keywords:** Phylogeography, *cox*1, 16S rRNA gene, *Rhipicephalus sanguineus*, *Rhipicephalus leporis*

## Abstract

**Background:**

*Rhipicephalus sanguineus* belongs to a complex of hard tick species with high veterinary-medical significance. Recently, new phylogenetic units have been discovered within *R. sanguineus*, which therefore needs taxonomic revision. The present study was initiated to provide new information on the phylogeography of relevant haplotypes from less studied regions of Europe and Africa. With this aim, molecular-phylogenetic analyses of two mitochondrial markers were performed on 50 ticks collected in Hungary, the Balkans, countries along the Mediterranean Sea, Kenya and Ivory Coast.

**Results:**

In the “temperate lineage” of *R. sanguineus*, based on cytochrome *c* oxidase subunit 1 (*cox*1) and 16S rRNA genes, *Rhipicephalus* sp. I was only found in the eastern part of the Mediterranean Basin (with relatively homogenous haplotypes), whereas *Rhipicephalus* sp. II occurred in the middle-to-western part of this region (with phylogenetically dichotomous haplotypes). Ticks identified as *R. leporis* (based on morphology and *cox*1 gene) were found in Kenya and Ivory Coast. These clustered phylogenetically within *R. sanguineus* (*s.l*.) (“tropical lineage”).

**Conclusions:**

In the Mediterranean Basin two mitochondrial lineages of *R. sanguineus*, i.e. *Rhipicephalus* sp. I and *Rhipicephalus* sp. II exist, which show different geographical distribution. Therefore, data from this study confirm limited gene flow between *Rhipicephalus* sp. I and *Rhipicephalus* sp. II, but more evidence (analyses of nuclear markers, extensive morphological and biological comparison etc.) are necessary to infer if they belong to different species or not. The phylogenetic relationships of eastern and western African ticks, which align with *R. leporis*, need to be studied further within *R. sanguineus* (*s.l*.) (“tropical lineage”).

## Background

*Rhipicephalus sanguineus* (*sensu lato*) (Acari: Ixodidae) belongs to a complex of at least 17 hard tick species, some with high medical and veterinary importance [1]. The type-species of this group was formerly called *R. sanguineus* (*sensu stricto*) (the brown dog tick), with cosmopolitan distribution owing to its high adaptability (i.e. being able to utilize endophilic and exophilic habitats in both urban and rural environment; having year-round activity and up to four generations per year; exhibiting passive or active host-seeking behaviour) [2]. Although typically a dog parasite (in all three developmental stages), *R. sanguineus* may also infest a wide range of domestic and wild animal host species, even humans. There are several animal and human pathogens (including zoonotic ones) that are or may be transmitted by *R. sanguineus* (reviewed in [3]). On account of its preference of warmer climates, global warming was predicted to induce the expansion of the geographical range of *R. sanguineus* [2].

According to current knowledge, *R. sanguineus* is not a single species. Molecular phylogeographic studies have found high intraspecific divergence of mitochondrial DNA between *R. sanguineus* from Brazil and Argentina, while a strong genetic relationship was detected between its European and Argentinean populations [4]. The differences between these strains were also demonstrated with morphological comparisons under scanning electron microscopy [5] and crossbreeding experiments [4]. Similarly, *R. sanguineus* collected in the USA and Mexico were shown to be genetically different [6]. A latitude-linked geographical pattern of the two major *R. sanguineus* groups has been confirmed by further, global scale studies, which showed with molecular-phylogenetic methods [7–9] or crossbreeding experiments [10] that (at least) two species might exist under this name, and both occur in the New World and in the Old World. These two clades have been designated as “tropical species” or northern lineage and “temperate species” or southern lineage [8, 11]. Moreover, a comprehensive morphological and phylogenetic study drew the attention to the existence of further operational taxonomic units (*Rhipicephalus* sp. I-IV) in addition to the “tropical species” [1]. The geographical distribution of these groups has recently been shown to be associated with climate variables, such as temperature [12].

In the above studies, certain regions of the globe appear to be underrepresented, as exemplified by several countries in or close to the Mediterranean Basin. Accordingly, it has been stated that further morphological and genetic studies of ticks in the *R. sanguineus* complex are needed from the Old World [1]. Thus, the primary aim of the present study was to provide relevant data from less studied regions, i.e. to report and compare in a phylogeographical context two mitochondrial markers of *R. sanguineus* from Hungary (where its occasional emergence can be anticipated; [13]), the Balkans and in a broader sense the Mediterranean Basin, as well as western and eastern Africa. In this way, representatives of *R. sanguineus* from both the “temperate” and “tropical” lineages have been included. The nomenclature of these categories is used *sensu* Dantas-Torres et al. [1] throughout the text.

## Methods

In this study, 68 ticks were collected (mainly from dogs) in 14 countries between 2010 and 2016. Eighteen ticks, morphologically identified as *R. sanguineus* and collected in France, Morocco, Algeria, Tunisia, Serbia and Turkey did not yield DNA, or their sequencing was not successful, therefore these samples were excluded from further study. Data of the remaining 50 ticks (collected in 11 countries) are shown in Table 1.

**Table 1:** Data of *Rhipicephalus* spp. ticks used in this study. The sex/stage of ticks and date of collection are not shown

Species^a^	Country	Location	Host of origin	*cox*1 sequence identity^b^	*cox*1 sequence accession number	Number of identical sequences	16S sequence accession number
“*R. sanguineus*”	Serbia	Kajtasovo	dog	630/630^2^	KX757879		KX793717
		Brnjica	dog	630/630^1^	KX757880		KX793718
		Jajinci	dog	629/630^1^	KX757881		KX793719
				630/630^1^	KX757882		KX793719
		Petnica	dog	629/630^2^	KX757883		KX793720
		Lebane	dog	629/630^1^	KX757885		KX793722
		Boka	dog	628/630^1^	KX757906		KX793738
				629/630^1^	KX757907		KX793738
				629/630^2^	KX757905		KX793739
	Croatia	Rovinj	unknown	630/630^2^	KX757887	2	KX793724
		Pula	dog	629/630^2^	KX757888		KX793725
				630/630^2^	KX757889		KX793726
		Zagreb	dog	629/630^2^	KX757890	1	KX793727
				630/630^2^	KX757896	2	KX793727
				628/630^3^	KX757893	2	KX793730
		Zadar	dog	629/630^2^	KX757892	2	KX793729
		Sibenik	dog	629/630^2^	KX757895		KX793731
	Romania	Babadag	golden jackal	630/630^1^	KX757915		KX793746
		Histria	dog	628/630^1^	KX757916		KX793746
	Hungary	Szekszárd^c^	dog	628/630^2^	KX757901	2	KX793734
	Malta	Siggiewi	dog	630/630^2^	KX757902	6	KX793735
	Italy	Piacenza	dog	630/630^2^	KX757904		KX793737
				626/630^3^	KX757903		KX793736
	Greece	Thessaloniki	dog	629/630^1^	KX757908	2	KX793740
	Algeria	Kehf Lagareb	bat (*Myotis punicus*)	630/630^2^	KX757910		KX793742
	Morocco	Al-Hoceima	dog	623/630^3^	KX757909		KX793741
	Ivory Coast	Abidjan	dog	620/620^4^	KX757914		KX793745
*R. rossicus*	Romania	Caraorman	dog	624/630^5^	KX757897		KX793732
		Lazuri	dog	628/630^5^	KX757898		KX793733
				630/630^5^	KX757899		KX793733
		Grindul	dog	629/630^5^	KX757900		–
*R. leporis*	Kenya	Turkana, Samburu	dog, cattle	627/630^6^	KX757911	4	KX793743
	Ivory Coast	Bas-Sassandra	dog	627/630^6^	KX757912	1	KX793744
				625/630^6^	KX757913	1	KX793744
				625/630^6^	KX757917	2	KX793744

^a^Currently species delineation within *R. sanguineus* (*s.l*.) requires revision [1]: here species names designating reference sequences in GenBank are used

^b^Number of nucleotides identical with reference sequence expressed as bp/bp. Superscript numbers indicate reference sequences (also shown on Figs. 2 and 3) as follows: ^1^KF219745; ^2^KU556745; ^3^AF081829; ^4^KF200084; ^5^JX394215; ^6^KM235720

^c^ *R. sanguineus* is not regarded as indigenous to Hungary; these specimens (collected in 2012 from a dog that has never left the country) exemplify rare autochthonous cases

**Fig. 1:**
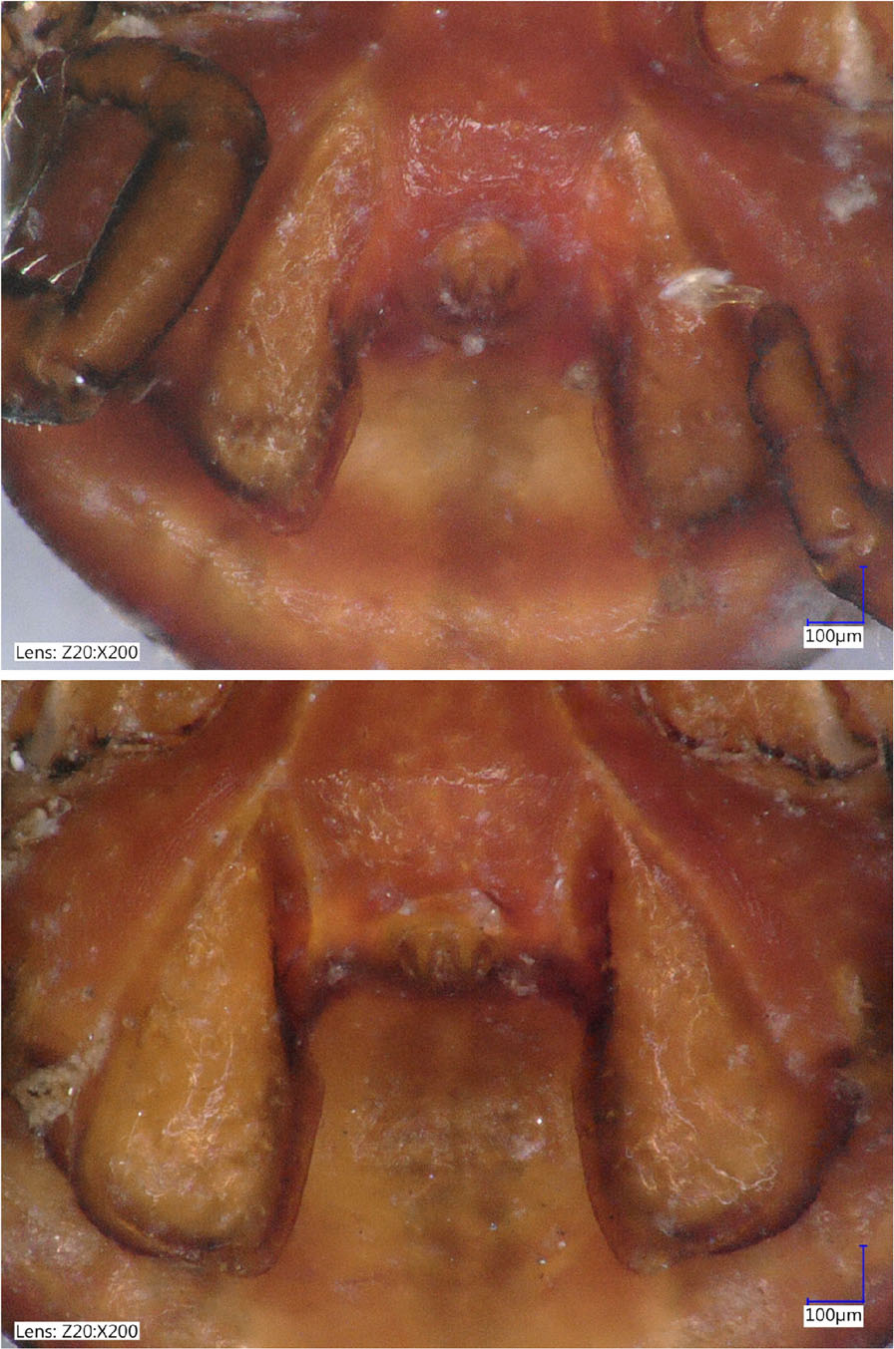
Adanal plates of two *Rhipicephalus* sp. I males (with only 1 bp difference in the amplified part of the *cox*1 gene; collected from the same dog in Jajinci, Serbia) showing similar shape (e.g. posteromedial corner), but highly different length-to-breadth ratio (upper: 2.7, lower: 2)

All ticks were stored in 96% ethanol. The morphology of ticks was preliminarily assessed using a stereo microscope (SMZ-2 T, Nikon Instruments, Japan, illuminated with model 5000–1, Intralux, Urdorf-Zürich, Switzerland) and standard keys (*R. sanguineus*: [14]; *R. rossicus*: [15]). In the category of *R. sanguineus* the adanal plate length to breadth ratio (which was reported to provide the only significant difference between *Rhipicephalus* sp. I-II: [1]) showed extreme variation even between conspecific males collected from the same dog (Fig. 1), therefore measurements were not taken. Identification of *R. leporis* males was based on the adanal and spiracular plates [16]. Pictures were made with a VHX-5000 (Keyence Co., Osaka, Japan) digital microscope.

DNA was extracted from ticks using the QIAamp DNA Mini Kit (Qiagen, Hilden, Germany), according to the manufacturer’s instruction, and including an overnight digestion in tissue lysis buffer with proteinase K at 56 °C. The cytochrome *c* oxidase subunit 1 (*cox*1) gene was chosen as the first target for molecular analysis, because of its suitability as a DNA-barcode marker for tick species identification [17]. PCR was modified from Folmer et al. [18] and amplified approximately 710 bp using the primers HCO2198 (5′-TAA ACT TCA GGG TGA CCA AAA AAT CA-3′) and LCO1490 (5′-GGT CAA CAA ATC ATA AAG ATA TTG G-3′) in a reaction volume of 25 μl, which contained 1 U (0.2 μl) HotStarTaq Plus DNA polymerase, 2.5 μl 10× CoralLoad Reaction buffer (including 15 mM MgCl_2_), 0.5 μl PCR nucleotide Mix (0.2 mM each), 0.5 μl (1 μM final concentration) of each primer, 15.8 μl ddH_2_O and 5 μl template DNA. For amplification, an initial denaturation step at 95 °C for 5 min was followed by 40 cycles of denaturation at 94 °C for 40 s, annealing at 48 °C for 1 min and extension at 72 °C for 1 min. Final extension was performed at 72 °C for 10 min.

To confirm the results obtained with *cox*1, another PCR was used to amplify approximately 460 bp of 16S rDNA of Ixodidae [19], using the primers 16S + 1 (5′-CTG CTC AAT GAT TTT TTA AAT TGC TGT GG-3′) and 16S-1 (5′-CCG GTC TGA ACT CAG ATC AAG T-3′). Other reaction components, as well as cycling conditions were the same as above, except for an annealing temperature of 51 °C.

PCR products were visualized in a 1.5% agarose gel. Purification and sequencing was done by Biomi Inc. (Gödöllő, Hungary). The sequences were submitted to the GenBank database under accession numbers KX757879–KX757917 (*cox*1) and KX793717–KX793746 (16S) (see Table 1). The MEGA model selection method was applied to choose the appropriate model for phylogenetic analyses. Phylogenetic analyses were conducted using the neighbour-joining method (p-distance model) and maximum likelihood method (Jukes-Cantor model) using MEGA version 6.0.

## Results

Out of the 50 molecularly analysed ticks, 38 were identified as *R. sanguineus* based on the amplified parts of their *cox*1 and 16S rRNA genes (i.e. corresponding to groups *Rhipicephalus* sp. I and *Rhipicephalus* sp. II of the “temperate lineage”, and *R. sanguineus* (*s.l*.) “tropical lineage”).

The *cox*1 sequences from *Rhipicephalus* sp. I had one to three nucleotides different among them (627–630/630 bp; 99.5–100% sequence similarity). In the subgroup *Rhipicephalus* sp. IIa, one to three nucleotides were different from each other (627–630/630 bp; 99.5–100% sequence similarity), whereas subgroup *Rhipicephalus* sp. IIb was more heterogeneous, with up to seven bp differences (623/630 bp; 98.9% sequence similarity). Comparisons between the two subgroups (a + b) of *Rhipicephalus* sp. II showed 28–32 bp differences among haplotypes, resulting in 94.9–95.6% (598–602/630 bp) sequence similarity. The *cox*1 sequences differed by 56–60 nucleotides between *Rhipicephalus* sp. I and II (570–574/630 bp; 90.5–91.1% similarity).

**Fig. 2:**
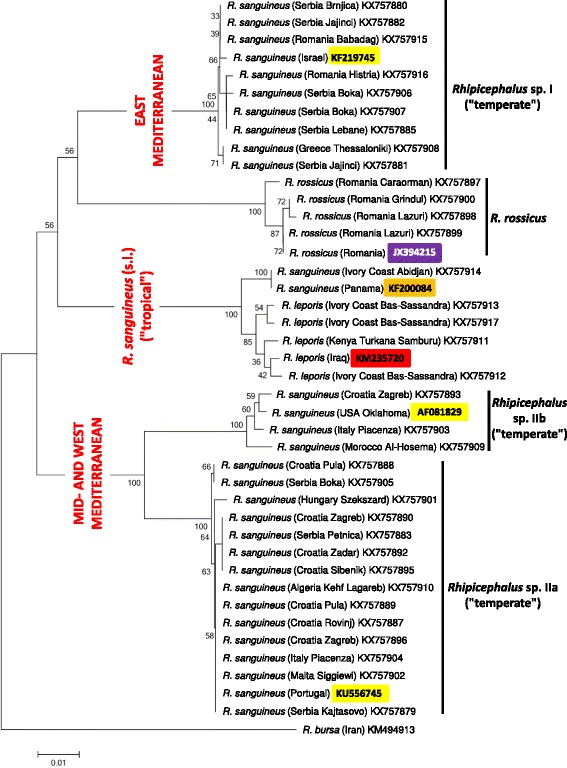
Phylogeny of *Rhipicephalus* spp. following neighbor-joining analysis of *cox*1 gene. For clarity, only one reference sequence (the closest *Rhipicephalus* haplotype available in GenBank from other studies) is included for each (sub)group. These reference sequences are indicated with coloured background of their accession numbers according to their taxonomic groups. Branch lengths represent the number of substitutions per site inferred according to the scale shown

**Fig. 3:**
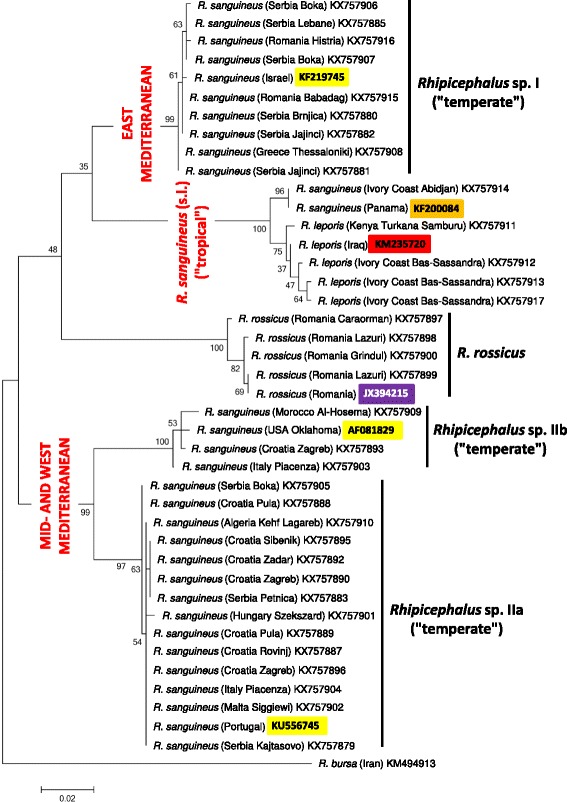
Phylogeny of *Rhipicephalus* spp. following maximum likelihood analysis of *cox*1 gene. For clarity, only one reference sequence (the closest *Rhipicephalus* haplotype available in GenBank from other studies) is included for each (sub)group. These reference sequences are indicated with coloured background of their accession numbers according to their taxonomic groups. Branch lengths represent the number of substitutions per site inferred according to the scale shown

**Fig. 4:**
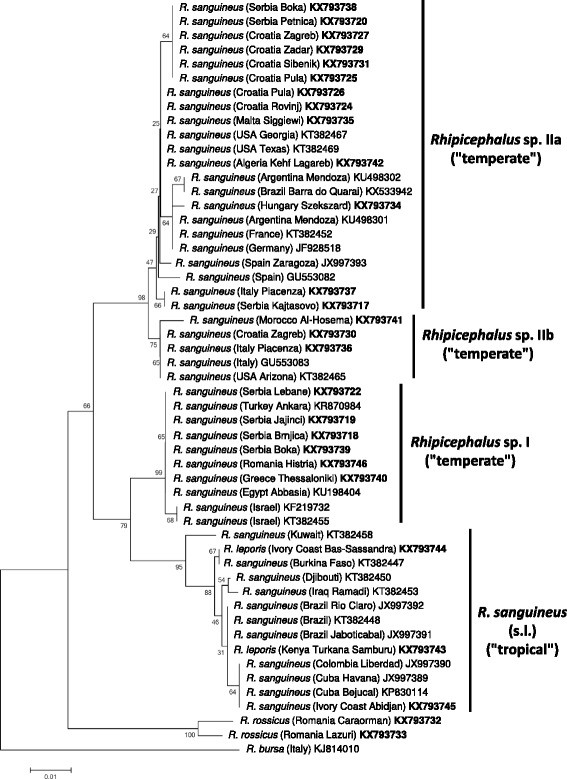
Phylogeny of *Rhipicephalus* spp. following neighbor-joining analysis of 16S rRNA gene. Sequences from this study are indicated with bold accession numbers. Branch lengths represent the number of substitutions per site inferred according to the scale shown

**Fig. 5:**
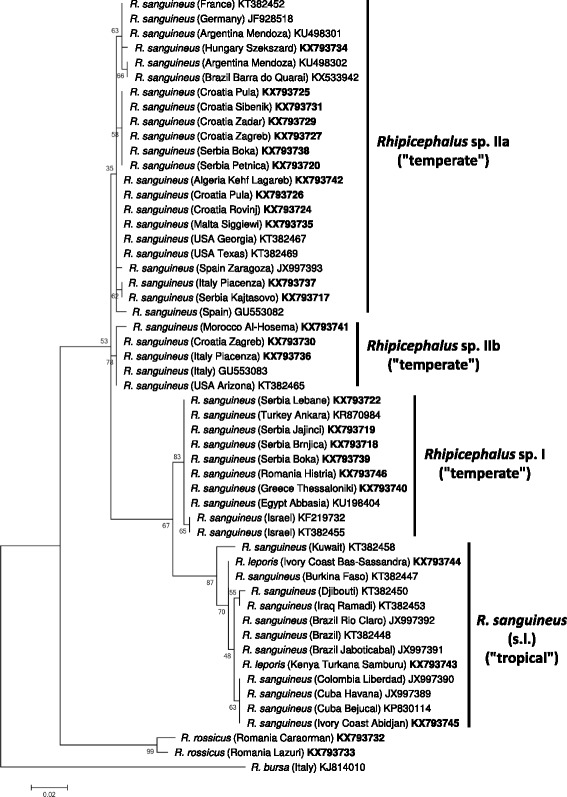
Phylogeny of *Rhipicephalus* spp. following maximum likelihood analysis of 16S rRNA gene. Sequences from this study are indicated with bold accession numbers. Branch lengths represent the number of substitutions per site inferred according to the scale shown

Phylogenetic analyses of *cox*1 sequence data indicated that haplotypes of *Rhipicephalus* sp. I formed a single clade (Figs. 2 and 3). On the other hand, representative sequences from *Rhipicephalus* sp. II formed two subgroups (a + b), with strong support (bootstrap = 99–100%) (Figs. 2 and 3). These relationships were confirmed following phylogenetic analysis of 16S rDNA sequence data, i.e. 16S rRNA gene haplotypes formed two main clusters (*Rhipicephalus* sp. I and II) within the “temperate lineage” (Figs. 4 and 5), although the separation of *Rhipicephalus* sp. IIa and IIb subgroups (based on these shorter sequences) was poorly supported in the maximum likelihood tree (bootstrap = 53%) (Fig. 5).

**Fig. 6:**
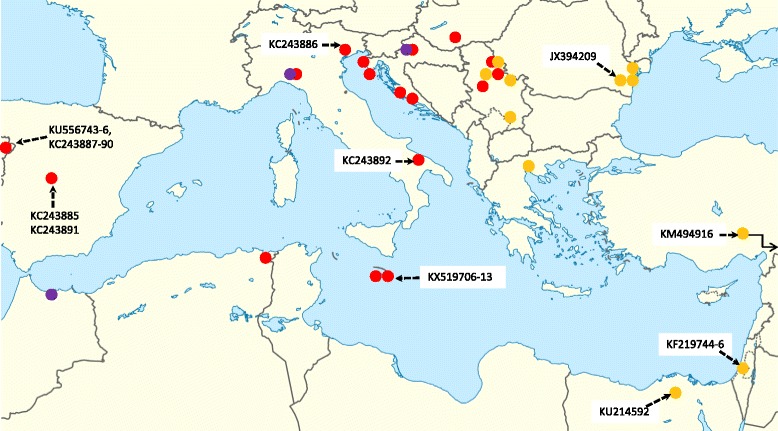
Geographical distribution of *Rhipicephalus sanguineus cox*1 haplotypes in and near the Mediterranean Basin. Coloured circles without accession number indicate samples from this study (*yellow*, *Rhipicephalus* sp. I; *red*, *Rhipicephalus* sp. IIa; *purple*, *Rhipicephalus* sp. IIb). Among these, multiple sequences for the same location are not shown. Haplotypes from other studies (which had 99–100% similarity with those in this study) are marked with GenBank accession numbers, connected with dash line arrows to the relevant location. Overlapping circles indicate the same location (where different haplotypes were found); the zigzag arrow marks the direction of location (outside this map) for the sample from Iran (Tehran). The location within a country is accurately indicated for samples of this study, as well as for Egypt, Italy and Romania from other studies, but for the rest of the samples only the country was known

Geographically, samples of *Rhipicephalus* sp. I. have only been collected in the eastern part of the Mediterranean Basin, similarly to the origin of other sequences from this group available in GenBank (Fig. 6). Complementarily to this, samples of the subgroup *Rhipicephalus* sp. IIa have been collected in the middle and western part of the Mediterranean Basin, as well as in Hungary, showing a zone of overlap with *Rhipicephalus* sp. I in Serbia. The geographical occurrence of *Rhipicephalus* sp. IIb was focal within the range of *Rhipicephalus* sp. IIa (in northern Morocco, Italy and Croatia-Zagreb; Fig. 6).

There was only one specimen from the Ivory Coast which was molecularly identified as *R. sanguineus* (*s.l*.) (“tropical lineage”). The *cox*1 sequence from this specimen was 100% identical to one sequence in GenBank, from Central America (Panama: KF200084; [20]).

**Fig. 7:**
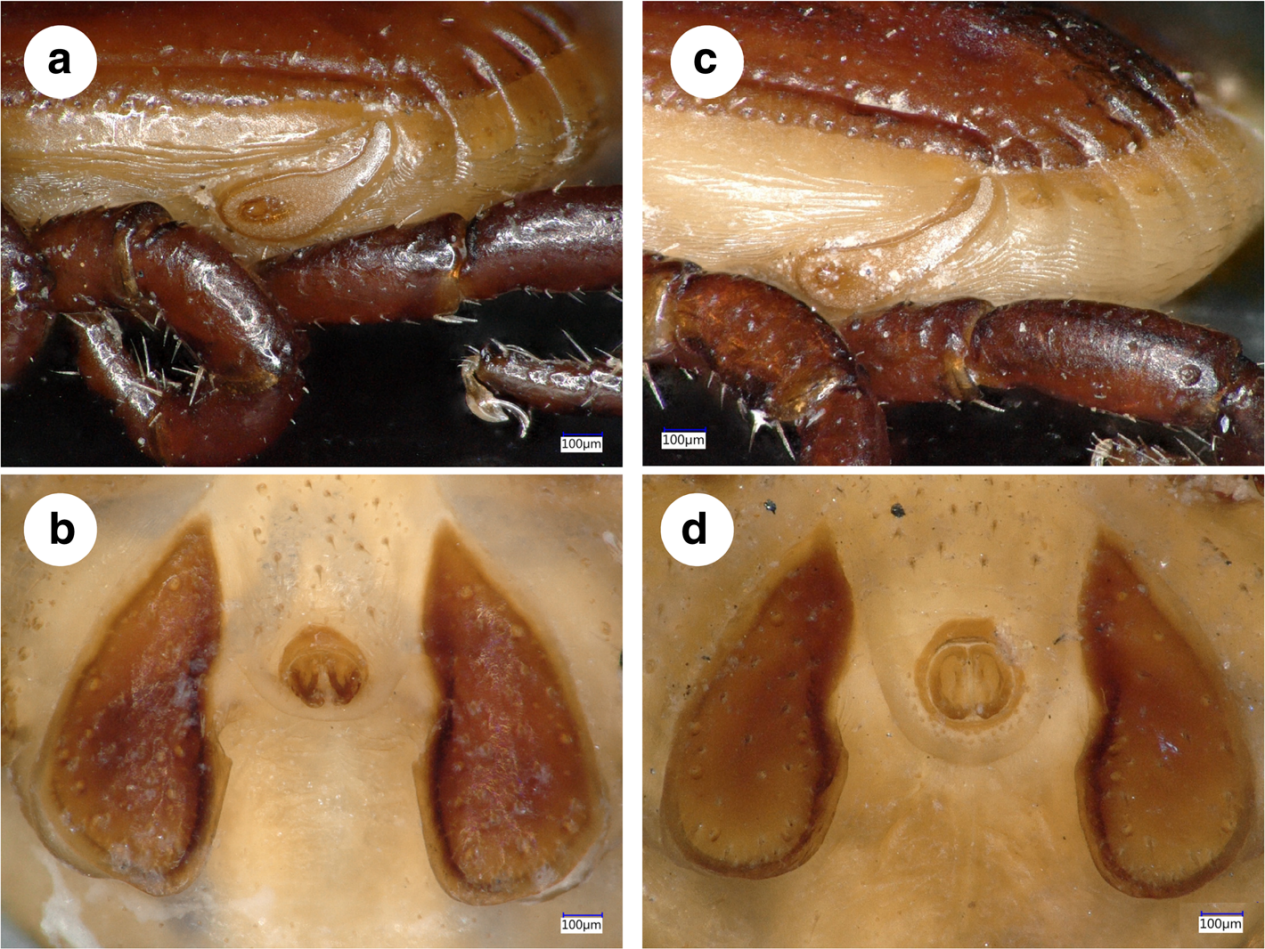
Diagnostically important structures of *R. sanguineus* and *R. leporis* males. **a** Left spiracular plate of *Rhipicephalus* sp. I collected in Histria, Romania. **b** Adanal plates of *Rhipicephalus* sp. II collected in Pula, Croatia. **c** Long and narrow dorsal prolongation of spiracular plates in *R. leporis*. **d** Tear-drop shaped, posteriorly rounded adanal plates in *R. leporis*

Based on *cox*1 gene, all remaining (eight) ticks from Kenya and the Ivory Coast (Table 1) clustered phylogenetically with *R. leporis* (Figs. 2 and 3). Males of *R. leporis* identified morphologically in the present study had very long and narrow dorsal prolongation of spiracular plates, and tear-drop shaped adanal plates rounded posteriorly (Fig. 7). Comparison of the *cox*1 sequences of relevant specimens with a voucher sequence in GenBank (Iraq: KM235720) confirmed them as *R. leporis* (624–626/629 bp; 99.2–99.5% similarity). The *cox*1 haplotypes of *R. leporis* and *R. sanguineus* (*s.l*.) (“tropical lineage”) showed ten bp differences (620/630 bp; 98.4% similarity) within the same country (Ivory Coast), and their separation was phylogenetically well supported (100%: Figs. 2 and 3).

In the 16S rRNA gene sequence analysis, the difference between *R. leporis* and *R. sanguineus* (s.*l*.) (“tropical lineage”) was not so evident. For instance, there was only one nucleotide difference between *R. leporis* isolates from east and west Africa (Kenya *vs* Ivory Coast). *Rhipicephalus leporis* from the Ivory Coast was not different from a *R. sanguineus* haplotype (KT382447) collected in neighbouring Burkina Faso, but both had five bp differences from *R. sanguineus* (*s.l*.) collected in Kuwait (394/399 bp; 98.7% similarity). Phylogenetically, *R. leporis* 16S haplotypes clustered separately from the latter (KT382458), but together with *R. sanguineus* (*s.l*.) isolates from the Old and New Worlds (Figs. 4 and 5).

*Rhipicephalus rossicus* was only identified in Romania (represented by four samples: Table 1). In the neighbor-joining analysis of *cox*1 sequences (Fig. 2) *R. rossicus* clustered as the sister group to eastern Mediterranean isolates (*Rhipicephalus* sp. I). However, based on 16S rRNA gene, *R. rossicus* formed a sister group to all *R. sanguineus* isolates (Figs. 4 and 5).

## Discussion

In a previous comprehensive study on the morphological and genetic diversity of *R. sanguineus* [1] it was shown that two groups under the “temperate lineage” (i.e. *Rhipicephalus* sp. I and *Rhipicephalus* sp. II) diverge molecularly to the point where they may be considered separate species. However, except for the adanal plate length-to-breadth ratio, no consistent morphological differences were observed between them [1]. Furthermore, in the present study the adanal plate length-to-breadth ratio was shown to vary even between almost identical haplotypes of *Rhipicephalus* sp. I. Therefore, for the investigation of their geographical distribution, haplotypes were here assigned to specimens of *R. sanguineus* based on molecular data, involving the analysis of two mitochondrial genetic markers. However, it also should be considered that analysis of relatively short sequences may cause an over-resolution of phylogenetic trees based on mitochondrial markers.

Phylogenetic analysis of *cox*1 sequences from new isolates of “*R. sanguineus*” in the present study confirmed the existence of paraphyletic groups previously referred to under the same species name [1]. Here it was also shown that in contrast to *Rhipicephalus* sp. I (which is a homogenous group), *Rhipicephalus* sp. II is rather heterogenous, consisting of two, phylogenetically well-defined clades (a + b). Phylogenetic analysis of 16S rDNA sequence data verified that these categories are also represented by formerly reported sequences from the New World: the clade composed of sequences classified as *Rhipicephalus* sp. IIa, based on phylogenetic analysis, included samples from Argentina, southernmost Brazil and the USA (Georgia, Texas), while another 16S rDNA sequence from the USA (Arizona) (as well as a *cox*1 sequence from Oklahoma) belonged to *Rhipicephalus* sp. IIb (Figs. 2, 3, 4 and 5).

The “tropical” and “temperate” lineages of “*R. sanguineus*” are reported to have a latitude related geographical pattern [1, 7]. Adding to this, it was demonstrated here that within the “temperate lineage” the distribution of *Rhipicephalus* sp. I and sp. II reflect longitudinal separation within and close to the Mediterranean Basin. Based on *cox*1 sequences, *R. rossicus* was demonstrated to phylogenetically cluster with the eastern Mediterranean *Rhipicephalus* sp. I, the former having an eastern distribution in Europe, emerging towards the west [15].

In this study the great majority of ticks from *Rhipicephalus* sp. I and *Rhipicephalus* sp. II mitochondrial lineages were collected in countries with a uniform Mediterranean climate (in Serbia haplotypes of these two categories even originated in the same location), and the phylogenetically separate subgroups *Rhipicephalus* sp. IIa and IIb occurred simultaneously in certain sampling sites (in Piacenza/Italy, Zagreb/Croatia). Therefore, the geographical patterns of *Rhipicephalus* sp. I, *Rhipicephalus* sp. IIa and *Rhipicephalus* sp. IIb in the Mediterranean Basin appear to be independent of current climatic conditions (unlike the geographical distribution of the “tropical” and “temperate” lineages of *R. sanguineus*: [12]).

If not current climatic conditions, then other factors influencing the tick life-cycle may provide a plausible explanation for the parapatric separation of *Rhipicephalus* sp. I and *Rhipicephalus* sp. II lineages in the Mediterranean Basin. Molecular evidence from a broad range of invertebrate and vertebrate taxa (i.e. potentially encompassing ticks and their hosts) indicate that southern peninsulas of Europe acted as major refugia during ice age(s), from which genetically distinct clades emerged [21]. While recolonization events to northern parts of Europe may have resulted in secondary sympatry for these clades, their genetic differences are still maintained and demonstrable. Thus, several (potential) host species of *R. sanguineus* had also been affected by glacial isolation in the same way. For example, wolf haplotype lineages and hedgehog species differ between Italy and the Balkans [21, 22], and genetically distinct populations of bank voles exist in the western and eastern Balkans [23]. These geographical patterns are similar to the one observed for *Rhipicephalus* sp. I and *Rhipicephalus* sp. II in the present study, suggesting that during ice age(s) the Mediterranean range of *R. sanguineus* (in sympatry with the above hosts) was not confluent, but inhabited by reproductively isolated tick populations. Nevertheless, successful interbreeding between ticks from *Rhipicephalus* sp. I and *Rhipicephalus* sp. IIb populations (listed as reference sequences from Israel and USA, Oklahoma on Figs. 2 and 3) had already been demonstrated [10].

*Rhipicephalus leporis* was hitherto known to occur in the Middle East and Central Asia [16], but here its specimens (identified both morphologically and genetically) are reported from Africa. Apart from a broad range of wild animals, dogs and goats are among the preferred hosts of this tick species [16]. Consequently, *R. leporis* could have been unknowingly transported on these hosts to regions outside its formerly known range, and not necessarily recently (considering the genetic divergence between its isolates from Iraq *vs* Kenya and the Ivory Coast). If confirmed, a likely explanation for *R. leporis* not being discovered in Africa until now is its morphological similarity to *R. sanguineus* (*s.l*.).

In the present study *R. leporis* and *R. sanguineus* (*s.l*.) (“tropical lineage”) clustered close to each other phylogenetically, with their *cox*1 sequences differing by 1.6%. This sequence divergence is within the range (i.e. 0.2–3%) of reported intraspecific nucleotide variation for the *cox*1 gene of *R. sanguineus* (*s.l*.) [1]. In addition, the *cox*1 sequence/phylogenetic difference between *R. leporis* and *R. sanguineus* (*s.l*.) (“tropical lineage”) was not reproducible with the analysis of 16S rRNA gene. When comparing 16S rDNA sequences of *Rhipicephalus* spp. it should be taken into account that the amplified part of the 16S rRNA gene was considerably shorter than *cox*1, and the average interspecific distance was reported to be lower for this gene than for either *cox*1 or 12S genes [17]. Therefore, the resolution of analysing these shorter 16S gene fragments may not suffice to distinguish closely related species. In addition, the sequence divergence between 12S gene sequences of *R. leporis* and *R. sanguineus* (*s.l*.) (“tropical lineage”) was reported to be of similar magnitude than between isolates of *R. leporis* or *R. sanguineus* (*s.l*.) themselves. For instance, sequences of the 12S gene of ticks from Kuwait, morphologically identified as *R. leporis*, were 99% similar to sequences of *R. leporis* from Iraq and *R. sanguineus* (*s.l*.) from South America [12]. Based on this ambiguity, further and larger scale studies may be needed to ultimately verify that *R. leporis* from east and west Africa are not intraspecific morphological variants of *R. sanguineus* (*s.l*.) within the “tropical lineage”.

## Conclusions

Two mitochondrial lineages within the “temperate species” of *R. sanguineus* (i.e. *Rhipicephalus* sp. I and *Rhipicephalus* sp. II) show different geographical distribution in the region of Mediterranean Basin, confirming limited gene flow between them. However, more evidence (analyses of nuclear markers, extensive morphological and biological comparison etc.) are necessary to infer if they belong to different species or not. Similarly, the phylogenetic relationships of eastern and western African ticks, which align with *R. leporis*, need to be studied further within *R. sanguineus* (*s.l*.) (“tropical species”).
